# Sintilimab in Patients with Previously Treated Metastatic Neuroendocrine Neoplasms

**DOI:** 10.1093/oncolo/oyac097

**Published:** 2022-06-01

**Authors:** Ru Jia, Yi Li, Nong Xu, Hai-Ping Jiang, Chuan-Hua Zhao, Rong-Rui Liu, Yue Shi, Yao-Yue Zhang, Shu-Yan Wang, Hui Zhou, Jian-Ming Xu

**Affiliations:** Senior Department of Oncology, The Fifth Medical Center of PLA General Hospital, Beijing, People’s Republic of China; Senior Department of Oncology, The Fifth Medical Center of PLA General Hospital, Beijing, People’s Republic of China; Department of Oncology, The First Affiliated Hospital of the Medical School of Zhejiang University, Hangzhou, People’s Republic of China; Department of Oncology, The First Affiliated Hospital of the Medical School of Zhejiang University, Hangzhou, People’s Republic of China; Senior Department of Oncology, The Fifth Medical Center of PLA General Hospital, Beijing, People’s Republic of China; Senior Department of Oncology, The Fifth Medical Center of PLA General Hospital, Beijing, People’s Republic of China; Senior Department of Oncology, The Fifth Medical Center of PLA General Hospital, Beijing, People’s Republic of China; Senior Department of Oncology, The Fifth Medical Center of PLA General Hospital, Beijing, People’s Republic of China; Medical Science and Strategy Oncology, Innovent Biologics, Inc., Suzhou, People’s Republic of China; Medical Science and Strategy Oncology, Innovent Biologics, Inc., Suzhou, People’s Republic of China; Senior Department of Oncology, The Fifth Medical Center of PLA General Hospital, Beijing, People’s Republic of China

**Keywords:** neuroendocrine neoplasms, checkpoint blockade, anti-PD-1 antibody, immunotherapy, neuroendocrine cancers

## Abstract

**Background:**

Neuroendocrine neoplasms (NENs) are a group of diseases that show high heterogeneity but have limited treatment options. This phase I study evaluated the safety and efficacy of sintilimab, anti-PD-1 monoclonal antibody, in treating advanced NENs.

**Methods:**

We prospectively enrolled patients pathologically diagnosed with NENs after standard treatment failure. Neuroendocrine neoplasms were classified into well-differentiated neuroendocrine tumors (NETs) and poorly differentiated neuroendocrine cancers (NECs). Every patient received sintilimab, and response was assessed every 9 weeks.

**Results:**

Twenty-four patients with a median age of 57.0 years were enrolled from November 2016 to 2017. The median Ki-67 index was 60%. Five patients had NET, 1 had NET G3, 17 had NEC, and 1 had mixed adenocarcinoma-neuroendocrine carcinoma. The most common primary tumor sites were the pancreas and gastrointestinal tract in 7 and 10 patients, respectively. In phase Ia trial, 2 patients received sintilimab 1 mg/kg every 2 weeks, one received 3 mg/kg every 2 weeks, and 21 patients enrolled in the phase Ib trial received 200 mg every 3 weeks. The objective response rate was 20.8% in all enrolled patients and 27.8% in NEC patients. The median progression-free survival was 2.2 and 2.1 months in patients with NET and NEC, respectively. The median OS was not applicable (NA) and 10.8 months (95% CI, 4.3, NA) with NET and NEC, respectively. The duration of response (DOR) was not reached, with a median follow-up time of 20.7 months. Treatment-related adverse events (TRAE) occurred in 17 (70.8%) patients. The most frequent TRAE was thyroid dysfunction (41.7%), and a grade 3 pulmonary infection occurred in 1 patient. The programmed cell death 1-ligand 1 (PD-L1)-positive (tumor proportion score ≥1%) rate was 18.8% (3 out of 16) and the expression of PD-L1 did not correlate with response.

**Conclusion:**

Sintilimab was well-tolerated and showed encouraging response in NECs.

**ClinicalTrials.gov Identifier:**

NCT02937116.

## Teaser

This phase I study evaluated the safety and efficacy of sintilimab, a monoclonal antibody PD-1 inhibitor, in treating advanced neuroendocrine neoplasms.

**Lessons Learned**

- Sintilimab demonstrated manageable safety and encouraging anti-tumor activity in patients with neuroendocrine cancer who had experienced disease progression following standard therapy, especially with tumors of gastro-esophageal origin.
- There is an urgent need to identify biomarkers of response.

## Discussion

**Figure 1.**
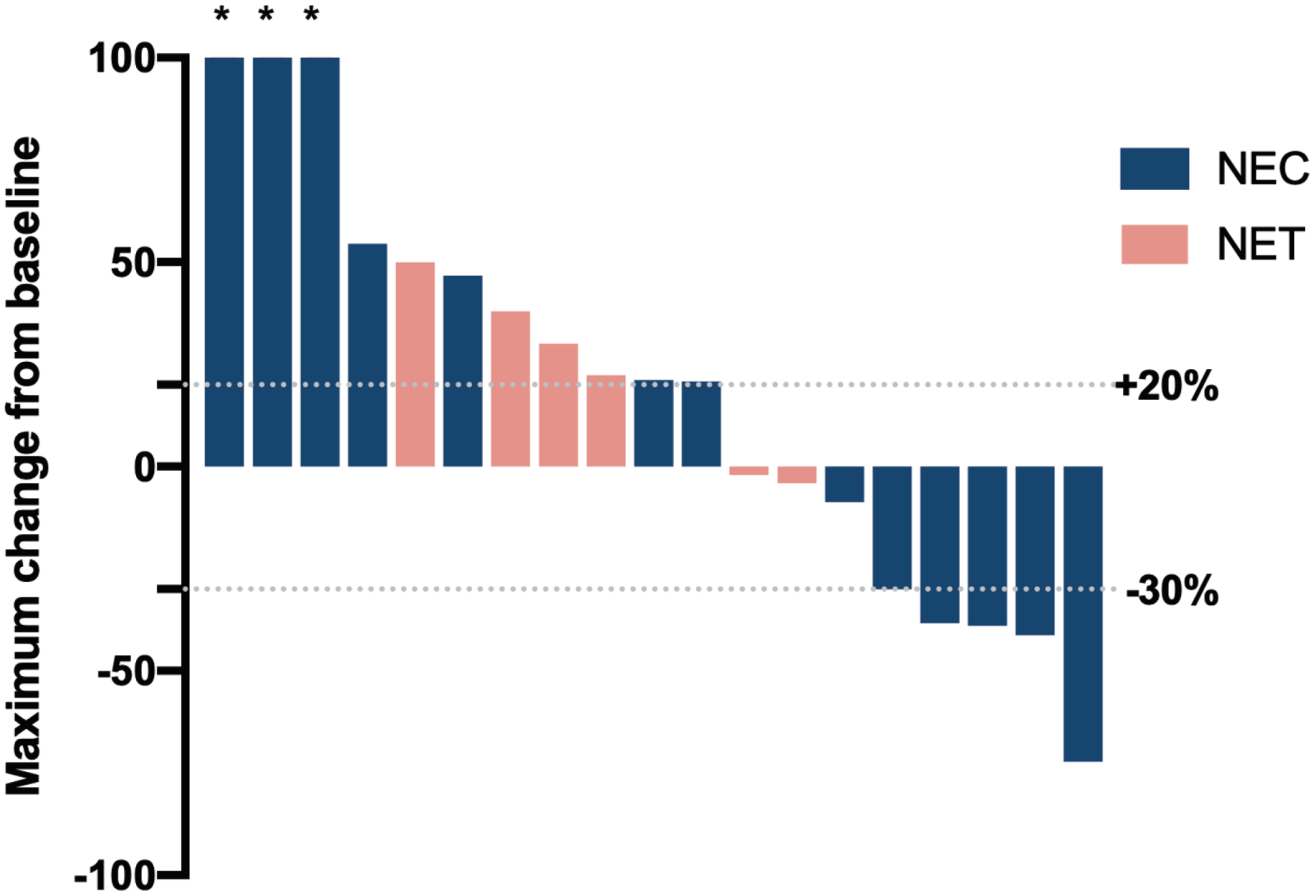
Maximum change from baseline in target lesion size assessed per RECIST v1.1 by investigator view with at least one postbaseline radiographic evaluation (*n* = 19). *Changes of more than 100% were truncated at 100%. Abbreviations: NEC, neuroendocrine carcinoma; NET, neuroendocrine tumor; RECIST, Response Evaluation Criteria in Solid Tumors.

The objective response rate (ORR) of patients with NEC (27.8%) in our study was comparable to previous second-line studies (29%-33%) of chemotherapy. Notably, the median DOR was estimated to exceed 1 year, much longer than historically observed in those treated with chemotherapy. However, no SD was observed in NEC in our trial, and 3 patients had rapid tumor growth of more than 100% at first evaluation (Figure 1), which resulted in a relatively short progression-free survival (PFS). Thus, the strategy of selecting patients with a cancer likely to respond is essential for those with NEC. The result with NETs was less encouraging. As seen in other studies, the anti-PD-1 antibody showed only minimal efficacy in those with slowly progressing NETs.

Further investigation revealed that 40% (2 of 5) patients with response had gastric NEC and the primary sites of all responding NECs were extra-pancreas, which was consistent with the published data of toripalimab treating NENs in China (Table 1). Both studies had an RR of 33% in gastro-esophageal NEC and 0 in pancreatic NEC. These 2 trials presented higher ORR and median OS compared with the Western studies reported in recent years. In the 2 Western trials that enrolled patients with G3 NEN, there was a much higher proportion of pancreatic NENs and a lower proportion of gastro-esophageal NENs. Moreover, the 2 Western trials enrolled some patients with well-differentiated NET that might have a lower RR to anti-PD-1 antibody. These factors might have resulted in different findings between Asian and Western studies.

**Table 1.** Comparison of efficacy of 4 G3 NENs’ trials.

	Sintilimab (*n* = 18)	Toripalimab (*n* = 32)	Avelumab (*n* = 29)	Pembrolizumab* (*n* = 21)
**G3 NENs, *n* (%)**				
Well-differentiated	0	0	10 (34.5)	NA
Poorly differentiated	18 (100)	32 (100)	19 (65.5)	NA
**Primary sites, n (%)**				
Pancreas	3 (16.7)	5 (15.6)	13 (44.8)	6 (28.6)
Gastro-esophagous	6 (33.3)	9 (28.1)	4 (13.8)	3 (14.3)
Intestine	3 (16.7)	11 (34.4)	3 (10.3)	5 (23.8)
other	6 (33.3)	7 (21.9)	9 (31.0)	7 (33.3)
ORR, *n* (%)	5 (27.8)	6 (18.7)	2 (6.9)	1 (4.7)
OS, months	10.8	NA	4.2	3.5

In this trial, 52.4% of patients were Ki-67 <55%.

Abbreviations: NENs, neuroendocrine neoplasms; NA, not applicable; ORR, objective response rate; OS, overall survival.

Four patients with NECs evaluated as immune unconfirmed PD (iUPD) continued treatment after first assessment of PD; 2 of these patients experienced further disease control for 4 and 10 months. In addition, the other 2 patients had tumors that appeared to have a slower growth rate after the first PD evaluation but assessed PD at next evaluation. Interestingly, these 2 patients were both alive at the data cutoff date with OS more than 23 months. This subgroup might not respond to anti-PD-1 antibody monotherapy but might benefit from combined therapy such as that with chemotherapy, targeted therapy, or other immunotherapies.

## Trial Information

**Table:** 

Disease	Neuroendocrine—other
Stage of disease/treatment	Metastatic/advanced
Prior therapy	At least 1 prior regimen
Type of study	Phase I, 3 + 3
Primary endpoints	Safety, tolerability
Secondary endpoints	
Investigator's analysis	Active and should be pursued further

## Additional Details of Endpoints or Study Design

The primary objectives of safety and tolerability of sintilimab in patients with NET were measured by the frequency of adverse events (AEs), treatment-related AEs (TRAE), AEs of special interest (AESI), and serious AEs (SAEs) and by monitoring laboratory abnormalities. The anti-tumor activity was the exploratory objective, which was measured as the ORR, time to response (TTR), duration of response (DOR), PFS, and overall survival (OS) using investigator-assessed tumor assessments according to Response Evaluation Criteria in Solid Tumors (RECIST) v1.1. PFS was defined as the time from the first dose of sintilimab to PD or death by any cause. Overall survival was defined as the time from the first dose of sintilimab to the date of death from any cause.

The expression of PD-L1 was measured using qualitative immunohistochemical assay using monoclonal mouse anti-PD-L1, clone 22C3 in formalin-fixed, paraffin-embedded (FFPE) tissue blocks or slides of biopsy or surgical specimens using EnVision FLEX visualization system with Autostainer Link 48. PD-L1 protein expression was determined using tumor proportion score (TPS), which is the percentage of viable tumor cells showing partial or complete membrane staining of any intensity.

## Drug Information

**Table:** 

Generic/working name	Sinitilimab, anti-PD-1-antibody
Company name	Innovent Biologics (Suzhou) Co. Ltd.
Drug type	Antibody
Drug class	Immune therapy
Dose	200 milligrams (mg) per flat dose
Route	IV
Schedule of administration	Patients with neuroendocrine neoplasms that failed to respond or became intolerant of standard treatment were enrolled to either the Phase Ia dose escalation study to receive sintilimab (a fully humanized anti-PD-1 monoclonal antibody, Innovent), or cohort B (digestive system cancer or neuroendocrine neoplasms) of the phase Ib trial to receive sintilimab 200 mg intravenously every 3 weeks. In phase Ia, “3 + 3” design was used during dose escalation: 1 mg/kg, 3 mg/kg, and 200 mg (1:1 randomization) and 10 mg/kg. After 28 days of dose limiting toxicity (DLT) observation, patients would repeat doses every 3 weeks for 200 mg level and every 2 weeks for the other dose levels. The selected dose for Ib was based on results of phase Ia and preclinical studies.

## Dose Escalation Table

**Table:** 

Dose level	Dose of drug: anti-PD-1-antibody	Number enrolled	Number evaluable for toxicity
1a-1	1 mg/kg every 2 weeks	2	2
1a-2	3 mg/kg every 2 weeks	1	1
1a-3	200 mg every 3 weeks	0	0
1a-4	10 mg/kg every 2 weeks	0	0
1b	200 mg every 3 weeks	21	21

## Patient Characteristics

**Table:** 

Number of patients, male	11
Number of patients, female	13
Stage	IV
Age: median (range)	57 (22.3-69.8) years
Number of prior systemic therapies: median (range)	2 (1-6)
Performance status: ECOG	0-7
	1-17
	2-0
	**3-0**
	**4-0**
Cancer types or histologic subtypes	Neuroendocrine carcinoma, 18; Neuroendocrine tumor , 16
Note	The primary tumor site of 10 patients was gastrointestinal and that of 7 was pancreatic. The other primary sites included liver (*n* = 2), lung (n=1), adrenal gland (*n* = 1), cervix (*n* = 1), and sacroiliac (*n* = 1). The median Ki-67 was 60%.

## Primary Assessment Method: All Patients (Exploratory)

**Table:** 

Number of patients screened	33
Number of patients enrolled	24
Number of patients evaluable for toxicity	24
Number of patients evaluated for efficacy	24
Evaluation method	RECIST 1.1
Response assessment, CR	0(0%)
Response assessment, PR	20.8%
Response assessment, SD	2(8.3%)
Response assessment, PD	14(58.3%)
Response assessment, Other	3(16.7%)
(Median) Duration assessments, PFS	2.1 months; CI 2.1-4.3
(Median) Duration assessments, OS	12.7 months; CI 5.8-NA
(Median) Duration assessments, Response duration	2.8 months
Duration of treatment	

Three patients were not assessed (PD [*n* = 2) and death [*n* = 1)]. Phase Ia enrolled 2 patients with NET evaluated as one SD and one PD, and one NEC evaluated as PR.

## Primary Assessment Method: NETs

**Table:** 

Number of patients screened	7
Number of patients enrolled	6
Number of patients evaluable for toxicity	6
Number of patients evaluated for efficacy	6
Evaluation method	RECIST 1.1
Response assessment, CR	0(0%)
Response assessment, PR	0(0%)
Response assessment, SD	2(33.3%)
Response assessment, PD	4(66.7%)
(Median) Duration assessments, PFS	2.2 months; CI 2.1-13.7
(Median) Duration assessments, OS	NA; CI 5.8-NA

## Primary Assessment Method: NECs

**Table:** 

Number of patients screened	26
Number of patients enrolled	18
Number of patients evaluable for toxicity	18
Number of patients evaluated for efficacy	18
Evaluation method	RECIST 1.1
Response assessment, CR	0(0%)
Response assessment, PR	5(27.8%)
Response assessment, SD	0(0%)
Response assessment, PD	10 (55.6%)
Response assessment, Other	3(16.7%)
(Median) duration assessments, PFS	2.1 Months; CI 2.0-4.3
(Median) duration assessments, OS	10.8 months; CI 4.3-NA
(Median) duration assessments, Response duration	2.8-NA

## Adverse Events (All Dose Levels, All Cycles)

**Table:** 

Name	^*^NC/NA	1	2	3	4	5	All grades
Thyroid dysfunction	59%	41%	0%	0%	0%	0%	41%
Hypoalbuminemia	76%	24%	0%	0%	0%	0%	24%
Aspartate aminotransferase increased	76%	24%	0%	0%	0%	0%	24%
Alanine aminotransferase increased	82%	18%	0%	0%	0%	0%	18%
Lipase increased	88%	6%	0%	6%	0%	0%	12%
Fatigue	88%	12%	0%	0%	0%	0%	12%
Hyperuricemia	88%	12%	0%	0%	0%	0%	12%
Hypothyroidism	88%	0%	12%	0%	0%	0%	12%
Cardiac disorders—other, T-wave abnormality	88%	12%	0%	0%	0%	0%	12%
Leukopenia	88%	0%	12%	0%	0%	0%	12%
Blood bilirubin increased	88%	12%	0%	0%	0%	0%	12%
Neutrophil count decreased	88%	12%	0%	0%	0%	0%	12%
Pulmonary infection	94%	0%	0%	6%	0%	0%	6%
Respiratory failure	94%	0%	0%	0%	6%	0%	6%
Pneumonitis	94%	0%	6%	0%	0%	0%	6%
Platelet count decreased	94%	6%	0%	0%	0%	0%	6%

The table lists all the treatment-related AEs that occurred in 17(70.8%) patients.

*NC/NA indicates no change or no adverse event. Percentage NC/NA plus percentage all grades total to 100%.

## Serious Adverse Events

**Table:** 

Name	Grade	Attribution
Pulmonary infection	3	Possible
Respiratory failure	4	Possible

One patient (4.2%) discontinued treatment permanently because of a grade 3 pulmonary infection resulting in respiratory failure.

## Assessment, Analysis, and Discussion

**Table:** 

Completion	Study completed
Investigator’s assessment	Active and should be pursued further

**Figure 2.**
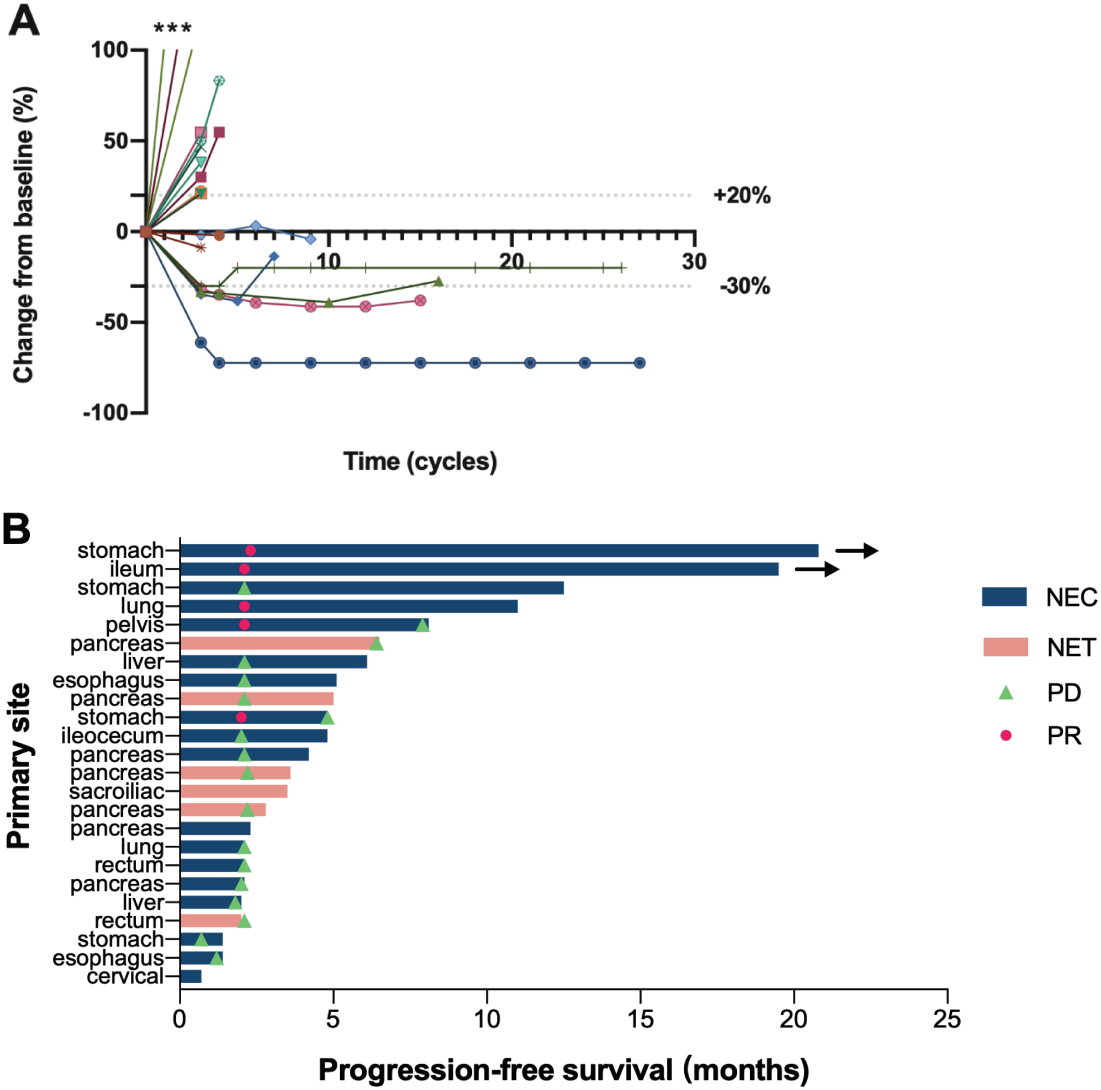
Antitumor activity of sintilimab in the total population. (**A**) Change from baseline of individual tumor burden in target lesion size (*n* = 19). *Changes of more than 100% were truncated at 100%. (**B**) Treatment exposure and duration of response assessed per RECIST v1.1 by investigator review (*n* = 24). Three patients were not assessed postbaseline as a result of clinical progression (*n* = 2) and death (*n* = 1). Two patients had new lesions without assessable RECIST changes and were assessed PD. The 2 black arrows indicate patients who were continuing treatment at the data cutoff date. Abbreviations: NEC, neuroendocrine carcinoma; NET, neuroendocrine tumor; RECIST, Response Evaluation Criteria in Solid Tumors; PR, partial response; PD, progressive disease.

Our results showed clinical meaningful anti-tumor activity in heavily treated NENs with an ORR of 20.8%. Different responses were observed in NET and NEC with an ORR of 0% and 27.8%, respectively (Figs. 1 and 2A). The safety profile of sintilimab was manageable and consistent with that of other anti-PD-1 antibodies. The most common AEs were low-grade (CTCAE grade 1 or 2) thyroid dysfunction and elevated aminotransferases. Treatment-related SAEs occurred only in one patient with pulmonary infection, which caused treatment, resulting in respiratory failure.

Sintilimab achieved a rapid and sustained response in 27.8% of NECs. Previous studies in NECs without response to standard first-line therapy reported an ORR of 33% for temozolomide-based therapy,^1^ 31% for FOLFIRI^2^ and 29% for FOLFOX.^3^ Since 44.4% of patients had been exposed to more than one line of prior therapy (Table 2), our study presented a comparable RR. Notably, the median DOR in patients who exhibited PR with sintilimab is estimated at over 1 year, much longer than that in those treated with chemotherapy (Fig. 2B).^3^ The prolonged DOR was consistent with other types of tumors who were treated with anti-PD-1 antibodies.^4^ The mOS of 10.8 months in patients with NECs was mostly driven by the durable response and the benefit of continuing treatment after PD. However, no SD was observed in NEC in our trial and 3 NECs had rapid tumor growth of more than 100% percent at first evaluation, which resulted in a relatively short PFS. Thus, a strategy of selecting those most likely to respond is essential for those with NEC.

**Table 2.** Baseline demographics and clinical characteristics.

	NENs (*n* = 24) *n* (%)	NETs (*n* = 6) *n* (%)	NECs (*n* = 18) *n* (%)
**Sex**			
Male	11 (45.8)	2 (33.3)	9 (50.0)
Female	13 (54.2)	4 (66.7)	9 (50.0)
Median age, years (range)	57.0 (22.3~ 69.8)	42.4 (27.0~ 62.0)	57.9 (22.3~ 69.8)
**ECOG PS score**			
0	7 (29.2)	3 (50.0)	4 (22.2)
1	17 (70.8)	3 (50.0)	14 (77.8)
**Primary tumor location**			
Gastrointestinal	10(41.7)	1(16.7)	9(50.0)
Pancreas	7(29.2)	4 (66.7)	3(16.7)
Liver	2(8.3)	0	2(11.1)
Lung	2(8.3)	0	2 (11.1)
Others^*^	3 (12.5)	1(16.7)	2(11.1)
Ki-67 (%), median	60	10	70
**Previous line(s) of chemotherapy**			
1	11 (45.8)	1(16.7)	10(55.6)
2	4(16.7)	2(33.3)	2(11.1)
3	6 (25.0)	2(33.3)	4(22.2)
≥4	3(12.5)	1(16.7)	2(11.1)
**Metastatic sites**			
Liver	12(50.0)	5(83.3)	7(38.9)
Lung	5(20.8)	2(33.3)	3(16.7)
Lymph nodes	16(66.7)	5(83.3)	10(55.6)
Bone	4(16.7)	1(16.7)	3(16.7)
Others	8(33.3)	3(50.0)	5(27.8)
**Previous treatment**			
Etoposide+platinum	18(69.2)	1(16.7)	17(94.4)
Everolimus	1(4.2)	1(16.7)	0
VEGFR-TKI	10(41.7)	5(83.3)	5(27.8)
Temozolomide	6(25.0)	3(50.0)	3(16.7)
Others	9(37.5)	3(50.0)	6(33.3)

Pelvis (*n* = 1), cervix (*n* = 1), and sacroiliac (*n* = 1).

Abbreviations: NENs, neuroendocrine neoplasms; NETs, neuroendocrine tumors; NECs, neuroendocrine carcinomas; ECOG PS, Eastern Cooperative Oncology Group Performance Status; VEGFR-TKI, vascular endothelial growth factor receptor tyrosine kinase inhibitors.

Further investigation revealed that 40% (2 of 5) patients with response had gastric NEC and the primary sites of all responding NEC were extra-pancreas, which was consistent with the recent published data of toripalimab treating NENs in China.^5^ In our study, the RR was 33% (2 of 6) in gastro-esophageal NEC, 33% (1 of 3) in intestinal NEC and 0 (zero of 3) in pancreatic NEC. In Lu’s study,^5^ the RR in gastro-esophageal, intestinal, pancreatic NEC was 33% (3 of 9), 0 (zero of 11), and 0 (zero of 5), respectively. Together, the 2 studies had an RR of 33% in gastro-esophageal NEC and 0 in pancreatic NEC, suggesting there could be a different biology between the 2 tumor origins.

It is notable that the 2 trials presented an ORR of 18.7%-27.8% and median OS of 10.8-NA months in NEC, compared with the Western studies reporting an RR of 4.7%-6.9% and OS of 3.5-4.2 months.^6-8^ As shown in Table 1, in the 2 Western trials that enrolled G3 disease, there was a much higher proportion of pancreatic NENs and much lower proportion of gastro-esophageal NENs. Moreover, the 2 Western trials enrolled some patients with well-differentiated NET, which appears to have a lower RR to anti-PD-1 antibody.^9^ These factors might have resulted in different findings between Asian and Western studies.

In general, studies on NETs with checkpoint inhibitors have been less encouraging than those of NEC. Western studies showed ORR of 3.7%–12% in NET and all patients who had responses in Keynote-158 had tumors that were PD-L1 negative.^9,10^ Although the DOR was relatively long (maximum 17.6 and not reached in the KEYNOTE-028 and KEYNOTE-158 studies, respectively), this result is confounded by slowly progressing disease. However, the toripalimab study showed opposite results in well-differentiated NET with an RR of 25.0% and a PD-L1-positive rate of 37.5%.^5^ Unfortunately, in the 6 patients with well-differentiated NETs enrolled in our study, 5 with available samples were all PD-L1 negative with a relatively low DCR of 33.3%. Thus, whether well-differenced NENs also can benefit from PD-1 antibody therapy is still under debate.

PD-L1 expression on tumor and immune cells has been associated with higher anti-tumor activity of PD-1 blockade in various tumors.^11,12^ PD-L1 expression was assessed in different cohorts of patients with NENs and several studies reported a significantly higher PD-L1-positive rate of 35.4%-100% in G3 GEP-NEN patients than only 0%-14.6% in G1/G2 patients.^9,13-15^ In addition, the expression of PD-L1 on tumor-infiltrating immune cells in G3 patients was relatively high.^13,16^ In our study, only PD-L1 expression on tumor cells was assessed and the positive rate was 0% in NETs and 37.5% in NEC, which is consisted with previously reported findings. The ORR was higher in patients with PD-L1-positive NEC than it was in those who were negative (66.7% vs 25.0%). However, because the number of patients with PD-L1-positive cancer was too small, the study lacked the power to distinguish the efficacy of the drug between PD-L1-positive and PD-L1-negative tumors.

In our study, patients were allowed to continue treatment according to the investigators’ assessment of their general condition and progression. Since standard immune-related evaluation was not published then, a 10% enlargement in diameter was used as cutoff of confirmed PD in the trial. According to established iRECIST guidelines,^17^ 4 NEC would be evaluated to have exhibited iUPD after first assessment of PD, consisting of 2 NEC each who experienced further disease control for 4 and 10 months. In addition, the other 2 NECs had tumors that nearly stopped growing after the first PD evaluation but then continued again and they subsequently experienced the next PD. Interestingly, these 2 patients were both alive at the data cutoff date with OS more than 23 months. This subgroup might not respond to anti-PD-1 antibody monotherapy but might benefit from combined therapy such as that with chemotherapy, targeted therapy, or other immunotherapies.

One limitation of the trial is its nonrandomized design and small sample size. The benefit of sintilimab monotherapy is not clear compared to standard chemotherapy or combination therapy (eg, with chemotherapy or targeted therapy) due to lack control groups. Neuroendocrine neoplasms are a variety of tumors with different biological behaviors, and NEC progresses rapidly with a high rate of comorbidities. It is necessary to validate efficacy in larger NENs population. The other limitation is that we did not explore other potential biomarkers such as CD8+ T-cell infiltration, tumor mutation load, and microsatellite instability (MSI) status that might influence the response to treatment.

## Data Availability

The data underlying this article will be shared on reasonable request to the corresponding author.
